# Network Compression as a Quality Measure for Protein Interaction Networks

**DOI:** 10.1371/journal.pone.0035729

**Published:** 2012-06-18

**Authors:** Loic Royer, Matthias Reimann, A. Francis Stewart, Michael Schroeder

**Affiliations:** 1 Bioinformatics, Biotec TU Dresden, Dresden, Germany; 2 Genomics, Biotec TU Dresden, Dresden, Germany; Institute for Research in Biomedicine, Spain

## Abstract

With the advent of large-scale protein interaction studies, there is much debate about data quality. Can different noise levels in the measurements be assessed by analyzing network structure? Because proteomic regulation is inherently co-operative, modular and redundant, it is inherently compressible when represented as a network. Here we propose that network compression can be used to compare false positive and false negative noise levels in protein interaction networks. We validate this hypothesis by first confirming the detrimental effect of false positives and false negatives. Second, we show that gold standard networks are more compressible. Third, we show that compressibility correlates with co-expression, co-localization, and shared function. Fourth, we also observe correlation with better protein tagging methods, physiological expression in contrast to over-expression of tagged proteins, and smart pooling approaches for yeast two-hybrid screens. Overall, this new measure is a proxy for both sensitivity and specificity and gives complementary information to standard measures such as average degree and clustering coefficients.

## Introduction

Over the last ten years, several experimental methods such as Yeast-two-hybrid (Y2H), affinity purification followed by mass spectrometry (AP/MS), and protein complementation assay (PCA) have been used for large-scale protein interaction mapping. Other approaches for reconstituting protein interaction networks range from computational and structural methods to manual curation and automated text-mining of large corpora of literature. Considerable obstacles have been encountered and the ways to assess data quality remain controversial. Despite many efforts, the interaction space for most species is still sparsely explored and reliable gold standards are difficult to define [1]. Consequently the problem of assessing the quality and coverage of protein interaction networks remains largely open. In this work we propose to quantify the richness in patterns and structure of different protein interaction datasets and investigate how this relates to quality.

### Large-scale interactome screens

Comparison of the first genome-wide Yeast Y2H networks by Uetz et al. and Ito et al. [2], [3], showed less than 20% overlap, which was slightly above random expectation. Shortly after, Gavin et al. performed one of the first large-scale screens using AP/MS [4]–[6]. Later screens by Gavin et al. and Krogan et al. were merged and filtered for false positives by Collins et al. [7]–[9]. Using a benchmark dataset, von Mering et al. reported that Y2H datasets had an estimated 1% coverage and 5% accuracy, whereas AP/MS methods had 35% coverage and 12% accuracy [10]. Up to date estimates put the accuracy of Y2H screens between 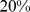 and 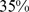 [11]. Recently, Tarassov et al. completed the first genome-wide in-vivo protein-fragment complementation assay (PCA) screen in yeast with positive predictive value of 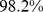 [12], and Yu et al. obtained a second-generation higher-quality high-throughput Y2H dataset [1]. Despite the large amount of interaction data obtained, there is only 63 interactions detected by both Y2H, PCA, and AP/MS screens in Yeast (see Fig. S9 and [13] for more details). The consensus is that different methods explore orthogonal sub-spaces of interactions with AP/MS favoring stable intra-complex interactions and Y2H transient inter-complex interactions [1], [13].

### Assessing quality

Several methods have been proposed for assessing the quality of protein interaction datasets. A first approach is to compare error-prone high-throughput data with interactions curated from literature on small-scale interaction studies [10], [14]. Indeed, manually curated interactions supported by multiple, independent pieces of evidence may be considered a gold standard [14], [15]. By recuration of a random sample of Human interactions mentioned in at least two publications, Cusick et al. recently reported that 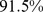 were correct [16], and Salwinski et al. showed that curation error rates are typically below 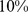 [17]. Several high-confidence datasets have been constructed by pooling information from literature curation and experimental data such as the ‘binary-GS’ dataset for Y2H [1], or the MIPS complex database for AP/MS [18]. A second approach consists in measuring the overlap between different datasets [10]. A third approach is to measure the proportion of interacting proteins that are co-expressed, functionally similar, co-localized in the cell, or phylogenetically related [1], [10], [19]–[22].

### More than the sum of its parts

The question of *quality* of protein interaction networks is a difficult and controversial one. It will likely be settled by intense experimentation using multiple and orthogonal methods [23]. In this work we propose instead to measure the network's informativeness by considering the statistically significant structures and patterns present in the networks. Consider a low sensitivity network consisting of isolated and 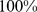 true interactions – each interaction is correct but no patterns emerge and complexes or pathways are impossible to identify. In contrast, consider a highly connected and noisy network with many spurious interactions – patterns occur, but not more often than expected by chance alone. Both networks share a lack of informativeness – either because of sparsity or because of too many false positives – but differ when considering the quality of individual interactions. Our working hypothesis is that true and complete interactome maps are not only characterized by the quality of their individual interactions but as well by the richness in overall structure. Because quality measures that assess the truthfulness of individual interactions are difficult to attain, our proposal is to complement these with a measure that evaluates the network structure as a whole. In the following we explain why we should expect the true networks to be rich in structure and how this can be quantified using network compressibility.

### Quantifying richness in patterns and structure

#### Modularity, redundancy and cooperativity imply compressibility

**Figure 1:**
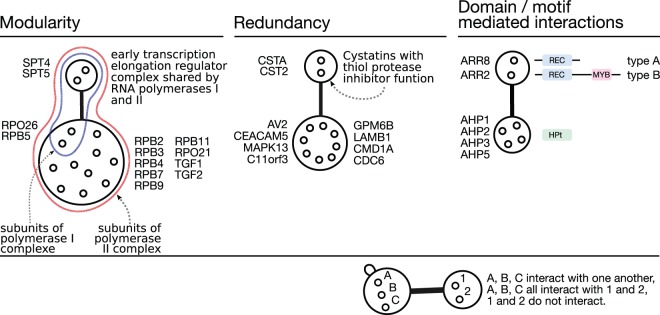
Modularity and redundancy in protein interaction networks. Modularity is a hallmark of protein interaction networks [7]. In the network by Collins et al. the proteins SPT4 and SPT5 have many common interaction partners [9]. It forms the SPT4/SPT5 sub-complex – shared by both the polymerase I and II [104] as well as complexes involved in mRNA capping and splicing [105]. Redundancy is seen, for example in the literature curated HPRD network [15], as proteins of same function sharing interaction partners – here two thiol protease inhibitors. Both modularity and redundancy of protein interactions can be explained by domain and motif mediated binding. For example, in Arabidopsis thaliana cytokinin-signaling pathway, the histidine kinases AHP1, AHP2, AHP3, AHP5 interact with response regulators ARR2 and ARR8 via their REC receptor domains [106].

Molecular systems in the cell are inherently modular, cooperative, and redundant [7], [24]–[26]. Fig. 1 shows that these properties are reflected in the networks – leading to compressible patterns of interaction. Similarly to the compressibility of genomic sequences due to the recurrence of similar sequences [27], [28], the compressibility of protein interaction networks is due to

- modules (e.g. protein sub-complexes which are re-used),
- redundant interactions (e.g. multiple inhibitors for the same enzyme),
- protein domain and motif mediated interactions [29] that form cliques and bicliques as shown in Fig. 1. Note that domain interactions do not necessarily lead to bi-cliques nor do bicliques nescessarily imply interactions between shared domains.

#### Entropy, compressibility, and Kolmogorov complexity

In computing, compression algorithms identify patterns in data and use these patterns to obtain compact representations, thus reducing data size. Lossless compression algorithms are reversible: the compressed representation is sufficient to recover the original data. In 1948, Shannon discovered a fundamental and unexceedable limit to lossless data compression based on the notion of entropy [30]. Entropy is intrinsically dependent on the pattern statistics of the data. Following this first insight, Kolmogorov and Chaitin later generalized this notion and introduced program-size complexity as the length of the shortest program needed to specify data. As put forward by Chaitin: “to comprehend is to compress” [31]. Chaitin's insight can be turned into an operational principle: compression algorithms can be used to analyze patterns and structure in data. For example, the information content of genomic sequences has been investigated in several studies [27], [28]. It was applied to alignment-free sequence comparison by conditional Kolmogorov complexity [32], and to protein sequence classification [33]. Similarly, there have been several attempts to quantify the information content of graphs.

#### Network information theory

There exist a variety of approaches for measuring the information content of graphs going back to Rashewsky et al. and Mowshowitz et al. who proposed to calculate the information content of graphs using Shannon's entropy formula [34], [35]. For example, Minoli et al. proposed to measure the *combinatorial complexity* of a network [36] and Jukna et al. defined graph complexity as the “minimum number of union and intersection operations needed to obtain the whole set of its edges starting from stars” [37]. More recently, a definition of network entropy based on topology configuration was used to segregate random network models [38]. And Dehmer at al. introduced a definition based on local vertex functionals for computing the entropy of chemical graphs and comparing chemical graphs [39], and another definition based on graph decompositions [40]. Graph entropy has also been used to characterize the resilience and robustness of protein interaction networks [41], [42]. Other approaches use network ensembles to evaluate entropy using concepts derived from statistical physics [43]. The general conclusion of these approaches when applied to complex biological networks is that these network topologies are markedly different from random graphs [44]. In general, there are suitable measures that quantify the difference between real and random networks. Protein interaction networks, for example, generally follow a power law degree distribution, which is markedly different from the degree distribution of a random network of the same size generated with the Erdös–Rényi model. As explained below, further insights into the information content of networks can be attained by quantifying network compressibility.

#### Evaluating information content with compression algorithms

Instead of measuring the network's information content using information theory and Shanon's entropy, we rely on the notion of graph compression. Other approaches for graph compression exploit neighborhood similarity, non-uniform network motif statistics, and scale-free properties of complex networks [45]–[52]. These algorithms rely on the idea that the more diverse the node neighborhoods are, the less compressible the network is, and the higher the network entropy is. For example, a network in which all nodes have nearly the same neighbors has a higher entropy whereas a network for which all nodes have different neighborhoods will have a higher entropy [53]. If two nodes in a network have nearly the same neighbors then they are also nearly exchangeable – to recover the original network few interactions need to be rewired. This implies that the amount of information necessary to encode both neighborhoods is less than the sum of that needed to encode each of them. This highlights the link between symmetry in networks and compressibility. The more symmetries a network has, the more compressible it is. Recently, [54] showed that ‘real-world’ complex networks are richly symmetric – much more than standard network models predict. Similarly, [55] showed that complex networks cluster in a tight region of the entropy-noise space. These result suggests that compressibility can be used to characterize complex networks. Network compressibility is then simply quantified by measuring data size before and after compression. In this work we use the power graph algorithm as a network compression algorithm [56].

#### Entropy, compressibility, and relative compressibility

Because we aim at comparing different networks, it is necessary to normalize against the effects of different sizes and topologies on a network's compressibility (see methods section for details and in depth discussion). Instead of measuring the entropy which varies according to the network's data size, we consider the *absolute compression rate* of the network defined as the proportion of edges removed after compression compared to the number of edges present before. For example, a compression rate of 70% means that among 100 edges in the original network, only 30 edges remain after compression. However, high connectivity networks with many edges per node are compressible solely because of the chance occurrence of patterns. To prevent this bias, we measure the compressibility of a network relative to a random ensemble of networks having the same number of nodes, edges and same degree distribution. We define the *relative compression rate* as the difference between the compression rate of a network and the average compression rate of these random networks (see methods section for details). For example, a network in which all proteins would be interacting with all other proteins has a relative compression rate of zero. Networks with no edges, a single edge, an arbitrary number of isolated edges, or a random high connectivity wiring of edges also have a relative compression rate of zero. In contrast, networks with statistically significant patterns can attain a relative compression rate as high as 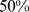. Furthermore, it should be noted that measuring the compressibility of very sparse and lowly connected networks is of limited interest – at the limit the compressibility for a network consisting of a single edge is meaningless. Throughout this work *compressibility* will implicitly refer to *relative compression rate*.

#### What is network quality

We understand network quality as encompassing both sensitivity and specificity. To illustrate this consider the following example: i) take a perfect and complete interactome and remove many interactions at random, or ii) take the same perfect and complete network and now add many interactions at random. As we will show, both alterations result in networks that have global properties closer to that of random networks and yet the truthfulness of individual positive interactions differs: individual interactions are more reliable in i) than in ii). The situation is reversed when looking at the network's complement, at the negative interactions: the absence of an interaction in i) is less reliable than in ii). Importantly, network quality is not solely determined by the quality of individual interactions.

In the following we give a four point validation of network compressibility as a measure of network's richness in structure – our proxy for the notion of overall network quality.

## Results

First we validate the link between relative compression rate and network quality as previously defined. We investigate to which extent it correlates with other measure proposed in the literature. We then compare the relative compressibility of all large-scale interactomes and discuss how assay parameters such as protein expression level, tagging, and pooling strategies influence the networks' relative compressibility. Importantly, we show that relative compressibility is independent of the network topology such as number of proteins, interactions, average number of interaction partners, or average clustering coefficient. Finally, we verify that networks derived from completely and accurately known complex systems are compressible at levels similar to the best interactomes.

### Validation 1 – False positives and false negatives decrease network relative compressibility

**Figure 2:**
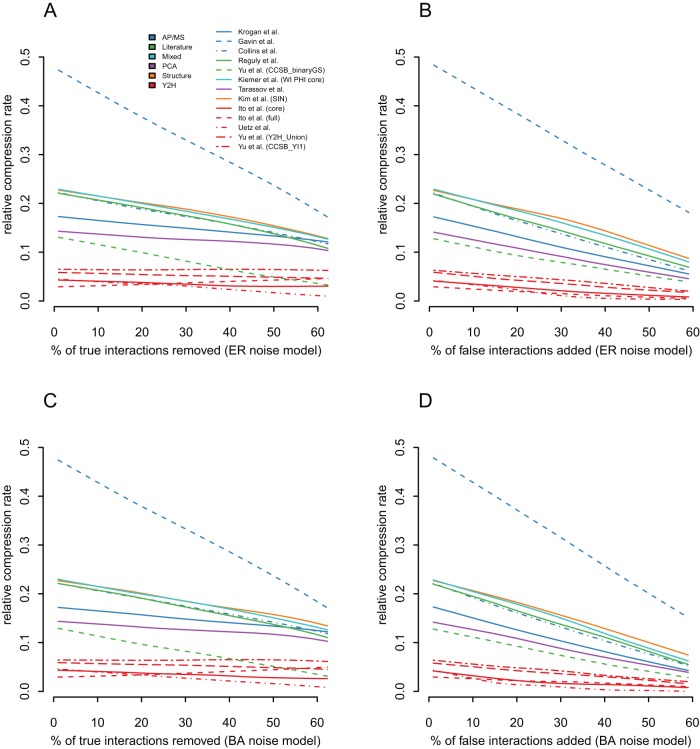
Effect of removal/addition of interactions on the relative compression rate. (**A and B**) Effect of removal/addition of interactions on the relative compression rate in 13 Yeast networks with the ER noise model. In order to validate the relationship between network quality and relative compressibility, we investigate the effect of false positives and false negatives on the relative compression rate for up to 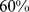 removed/added interactions with the ER models. Independently of the experimental system or network topology, both false positives and false negatives consistently reduce the relative compression rate when the proportion of added or removed interactions is increased. (**C and D**) Effect of removal/addition of interactions on the relative compression rate in 13 Yeast networks with the BA noise model. Inspired by the BarabÃ¡si-Albert preferential attachment model of network growth, we investigate the effect of false positives and false negatives biased towards highly connected proteins and lowly connected proteins, respectively. Therefore, the scale-free network topology is preserved and “interaction-rich proteins get richer and interaction-poor proteins get poorer”. As for the random (ER) noise model, we observe that independently of the experimental system or network topology, both false positives and false negatives consistently reduce the relative compression rate. While both models give similar curves, the BA model decreases the relative compression rate by an additional 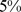 for high noise levels (60%).

If relative compressibility measures the fidelity of the networks to the systems they represent, then the relative compression rate should deteriorate with the addition of noise to networks. Noise can be applied by randomly adding interactions – introducing false positives (FP) – or by randomly removing interactions – introducing false negatives (FN). We consider two models for adding or removing interactions in protein interaction networks. In the Erdös–Rényi model (ER), the choice of interactions is independent of the network topology and all possible interactions are equally likely to be selected for addition or removal [57]. In contrast, in the Barabsi-Albert model (BA), the scale-free topology is preserved [58]. It is assumed that false positives are more likely for highly connected proteins (“the rich get richer”) while false negatives are more likely for poorly connected proteins (“the poor get poorer”). This gives a total of four combinations: FN/ER, FP/ER, FN/BA, FP/BA which were applied to 13 Yeast networks (5 Y2H, 3 AP/MS, 1 PCA, 2 literature, 1 structure) adding and removing up to 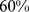 of interactions (see Methods for details). As shown in Fig. 2, we find that false positives and false negatives decrease the relative compression rates of networks – independently of the system from which the network is derived and independently of the model considered for false positives and false negatives. Thus, low sensitivity and low specificity implies low relative compression rate. Furthermore, relative compressibility decreases linearly with the increase of noise. For example, for the Collins network, each additional 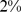 of false positives or false negatives leads to a 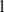 percentage point decrease in relative compressibility.

Networks that have a low relative compressibility (below 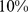) are proportionally less affected by noise. For example, *CCSB YI1* [1] remains stationary at around 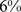. There is one exception, Ito full is the only network to increase in compressibility when interactions are removed – we examine this network in the next paragraph.

#### Why does removing interactions from Ito full slightly increases its network compressibility?

With one exception (Fig. S2), all curves in Fig. 2 decrease with the addition or removal of random interactions. The Ito full dataset consists of both Ito core interactions as well as unreliable interactions that were observed only once [59]. We observe that removing interactions from Ito full brings its relative compression rate to levels comparable to Ito core without noise. This is explained by an increase of the signal to noise ratio in the network. The only case in which removing interactions from a network increases its compressibility is when many removed interactions are isolated and not part of structures in the network. Notice that this effect occurs only when *removing* interactions and not when adding false positive interactions (Fig. 2BD). Comparing the structures of both networks we found that 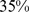 of Ito full interactions are incompressible, whereas this number is 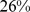 for Ito core, and for example 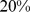 for Binary-GS. The high proportion of incompressible interactions in Ito full – many of which are probably false positives – explains this effect. However, because of the high variance when computing the effect of noise on lowly compressible networks (below 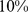), the small increase of Ito full must be interpreted with caution (see high variability in Fig. S2 requiring LOWESS filtering).

#### Effect of random removal or addition of proteins on the relative compression rate

**Figure 3:**
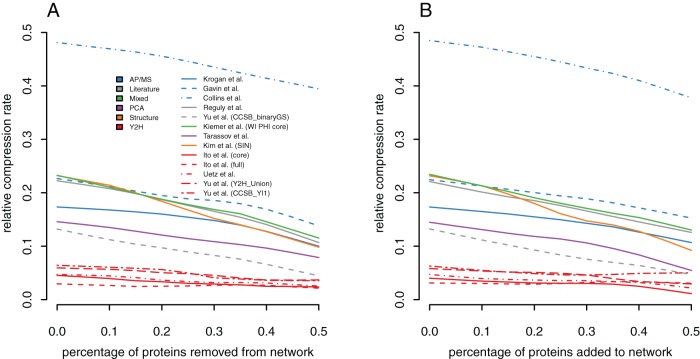
Influence of under-sampling and ‘over’-sampling on the relative compression rate. (**A**) The relative compression rate decreases slowly when proteins – and all their interactions – are removed from networks (compare to Fig. 2). For example, removing half of the nodes in the Collins network decreases its relative compression rate by just 10 percentage points. This shows that the effect of under-sampling is not as strong as the effect of false positives and negatives. (**B**) We also investigate the effect of the addition of proteins and corresponding interactions to the network (see materials and methods for details).

In addition, we consider false positives and false negatives caused by missing or added proteins. *Under-sampling* in screens is the main cause for missing proteins. We removed up to 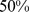 of proteins from the same 13 yeast networks and observed that the relative compressibility decreases with protein removal (Fig. 3A, see Methods for details). We also added up to 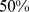 extra proteins to the networks while preserving the original network degree distribution. We also observe a decreases in relative compressibility. Indeed, the relative compression rate only increases if the added protein has *exactly* the same interaction profile as another protein already in the network. This case happens rarely when proteins are chosen randomly. Therefore, the net effect is increased randomness in the network and therefore a decrease of relative compressibility (Fig. 3B). In general, a strong effect is not expected since the patterns exploited by power graph analysis – non-trivial cliques and bicliques – are robust to random node removal or addition. Indeed, when comparing these results to those of Fig. 2 we note that removing or adding proteins and all their interactions has a lesser influence on the relative compressibility than the independent removal or addition of interactions.

### Validation 2 – Relative compression rates correlate with published interaction confidences

**Figure 4:**
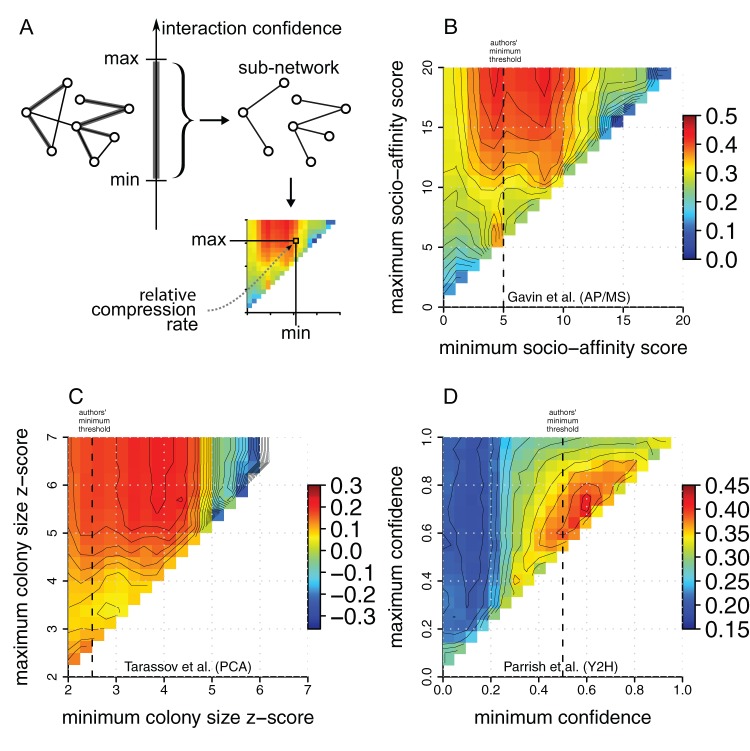
Correlating interaction confidence scores with relative compressibility. (**A**) Measuring the relative compression rate of sub-networks obtained by slicing networks for different ranges of confidence scores. The color of each cell indicates the relative compression rate of each sub-network and the vertical dotted lines indicate the authors' choice of minimum confidence thresholds. (**B**) For Gavin's network we observe that the sub-network with the most interactions and the highest relative compression rate is found for a minimum socio-affinity score of 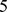 and a maximum of 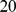. This is in agreement with the minimum of 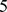 recommended by Gavin et al. – interactions with a lower score have reproducibility of less than 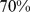 [7]. (**C**) For Tarassov's network we find that the highest relative compression rates are found for a minimum z-score of 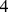 and a maximal z-score of 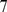. However, lower confidence interactions do not significantly decrease the relative compressibility of the sub-networks – at most 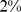 relative compressibility points are lost when including lower confidence interactions (z-score from 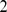 to 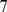). This is in agreement with the relatively generous threshold of 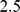 used by the authors on the colony size z-score. (**D**) Parrish's network we observe low relative compressibility for sub-networks containing low confidence interactions (minimum 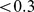). In contrast to the Gavin and Tarassov networks, the highest relative compression rate is not found when including high confidence interactions. Instead, it is found for a sub-network with confidences between 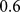 and 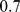 which agrees with the author's threshold of 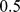 between high and low quality interactions.

Published interactomes are often reported as binary interactions, i.e. either two proteins interact or not. Underlying these data are confidence scores – authors define a threshold and only report interactions above that threshold. Defining such a threshold is a difficult compromise since a conservative threshold may improve precision but lowers the coverage, while a generous threshold achieves the opposite effect. Thus, the threshold controls the amount of false positives and false negatives in the network and the question arises of how is this reflected in the compression rates. To answer this question we systematically analysed the networks of Gavin (TAP/MS), Tarassov (PCA), Parrish (Y2H), Kiemer (WI-PHI integrated network) and computed the compression rates for networks defined by interactions above a minimum and below a maximum confidence score (see Fig. 4A and Methods for details). The results for all three networks are given in Fig. 4 and Fig S1. First, we note that complete networks – lowest minimum and highest maximum – are not necessarily the most compressible. Second, with the exception of the network by Parrish, the most compressible sub-networks include the interactions of highest confidence. Moreover, including interactions of low confidence consistently decreases the compressibility of the corresponding sub-networks.

#### Gavin network (TAP/MS)

Remarkably, for Gavin's network, the highest relative compression rate is found for a minimum confidence score (socio-affinity index) of 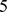 – a threshold recommended by the authors. We also observe the detrimental effect of both false negatives and false positives when imposing excessively high minimum or low maximal thresholds to the data: keeping only interactions with a score above 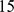 leads to similarly low relative compression rates as keeping only interactions with a score below 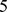.

#### Tarassov network (PCA)

For Tarassov's network we find that the highest relative compressibility is found for a minimum score of 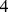 and a maximal score of 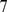. However, most sub-networks with high maximum thresholds have similar compressibility (between 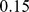 and 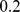) unless the minimum threshold is set too high (above 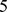). In agreement with this observation the authors choose to include most lower confidence interactions with a minimum threshold of 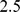. Interactions with a score above 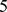 form less network motifs and thus the sub-networks are lowly compressible. Yet, including these interactions together with interactions with a score above 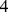 gives more compressible sub-networks than without – indicating that these interactions belong to structures formed for slightly lower confidences.

#### Parrish network (Y2H)

For Parrish's network we observe that interactions with confidence scores below 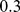 form sub-networks with low relative compression rates. In particular, we find that the sub-networks with lowest relative compression rates are found for a minimum of 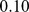 and maximums below 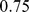 – which indicates that interactions with a confidence around 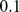 are detrimental to relative compressibility. This is in agreement with the analysis by Parrish et al. which shows that interactions with a confidence of about 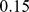 have the highest proportion of false positives [60]. This is estimated from a training set of likely true positives and true negatives – see Fig. 2A in [60]. Moreover, the peak in relative compression rate is found for a minimum threshold of 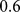, in agreement with the author's confidence threshold of 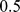 separating high from low confidence interactions. The value of 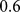 is in fact closer to the confidence score for which functional homogeneity between interacting proteins becomes significant – see Fig. 2C in [60]. Surprisingly, interactions of *very* high confidence (above 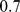) are detrimental to the relative compressibility. These high confidence interactions do not fit together with the other high-confidence interactions (above 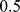). Either the confidence scores are flawed for very high values, or network compressibility is inapplicable in this case. Our suspicion is that erroneously high Leu and *LacZ* reporter activities – used for deriving the confidence scores – could be responsible. Supporting this hypothesis and following intuition is the observation that for the Gavin, Tarassov, and the WI-PHI networks, raising the upper confidence bound always increases compressibility.

#### WI-PHI network

We also tested a high quality merged dataset: the WI-PHI network [61]. WI-PHI is a yeast interactome enriched for direct physical interactions compiled from several datasets (Gavin, Krogan, Ito, Uetz, and BioGRID, BIND, IntAct, Mint) [2], [7], [8], [59], [62]–[65]. The authors computed socio-affinity scores for all interactions covering 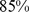 of the yeast proteome. Similarly to the networks by Gavin and Tarassov, excluding low-confidence interactions (score below 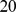) leads to a higher compressibility than is achieved for the whole network. The authors defined the *core* dataset as all interactions with a score above 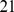. Our analysis confirms that interactions with score below 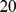 are detrimental to the compressibility (Fig. S1).

### Validation 3 – Author's gold standard datasets have highest relative compression rate

The network by Collins et al. is a merge and re-analysis of the raw data from the Gavin and Krogan datasets aimed at improving coverage and reducing false positives [9]. We observe that this dataset has a higher relative compression rate (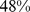) than both original datasets interpreted with the plain *spoke* model (Gavin 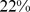 and Krogan 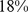). This is in agreement with the author's assessment which showed that their consolidated dataset has a higher functional homogeneity than the Gavin or Krogan datasets – see Fig. 2 in [9].

Yu et al. compared their novel experimental dataset (CCSB-YI1) and their own merge of several datasets (Y2H-Union) to a gold standard of binary interactions derived from literature (CCSB-binaryGS) [1]. We find that this recent gold standard dataset has a higher relative compression rate (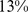) than all Yeast Y2H datasets.

Ito et al. discouraged the use of the Ito full dataset and instead recommended the use of a subset: Ito core. We observe that the Ito core network has a slightly higher relative compression rate (of 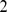 percentage points) [59]. Since Ito full has the same if not a greater coverage than Ito core, we can assume that the difference in relative compression rate is attributable to false positives.

Similarly, false positive estimates by Lemmens et al. [11] correlate with relative compressibility: the Stelzl dataset achieved the highest MAPPIT-retest success rate of 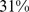 and also has a higher relative compression rate (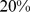) compared to the datasets from Rual (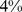), Yu (CCSB-YI1, 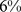), and Simonis [66] (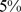) – see Fig. 2 in [11].

Finally, the WI-PHI core network by Kiemer et al. [61], enriched for direct physical interactions has the highest relative compression rate of all yeast networks when excluding socio-affinity Gavin and Collins networks. It should be noted that these ‘gold standard’ networks have compressibility rates within a large range of 13%−50% which suggests that they do not necessarily have the same level of quality. Networks are deemed high-quality in a specific experimental context. There may well be differences in quality between these networks for which we dont have independent evidence. The difficulty here is the absence of reliable and undisputed gold standards or means to compare different proposals for gold standards.

### Validation 4 – Compressibility correlates with co-expression, co-localization and shared function

**Figure 5:**
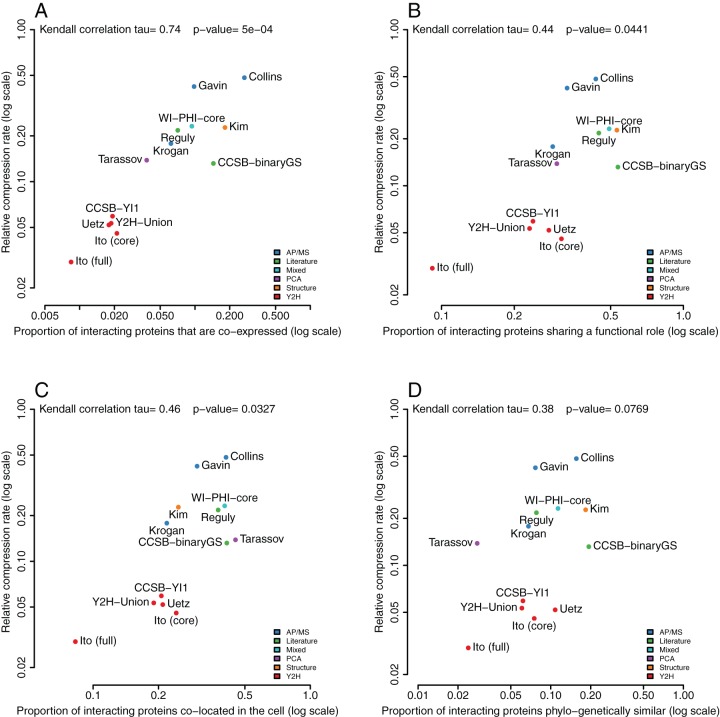
Correlation of the relative compression rate with gene co-expression, functional similarity, cellular localization, and phylogenetic profile similarity for 12 Yeast networks. For all interacting pairs of proteins for which we have information about both, we compute the proportion – or assortativity ratio – of interacting proteins that are significantly co-expressed, share a cellular function, are found in at least one common cellular compartment, and have similar phylogenetic profiles. We normalize these ratios by subtracting the average proportion found for equivalent randomized networks similarly to the relative compression rate (see Methods for details). (**A**) Relative compression rate versus relative proportion of interacting proteins that are co-expressed. The Kendall correlation (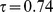) is the highest of all four studied correlations. (**B**) Relative compression rate versus relative proportion of interacting proteins that share at least one functional role. (**C**) Relative compression rate versus relative proportion of interacting proteins that share at least one cellular localization. (**D**) Relative compression rate versus relative proportion of interacting proteins that have similar phylogenetic profiles. The low Kendall correlation (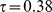) and poor 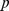-value (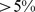) indicates a poor correspondence between relative compression rate and shared evolution.

Assortativity in protein interaction networks refers to the preference of proteins to interact with other proteins that are similar or share certain properties [67]. It has been previously proposed as a means of evaluating network quality when applied to gene co-expression, functional similarity, cellular localization, and phylogenetic profile similarity [10]. Fig. 5A shows that the relative compression rate is highly correlated to the proportion of co-expressed gene pairs corresponding to interacting proteins (Kendall 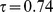). There is a weaker correlation with function (Fig. 5B, 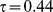) and with co-localization (Fig. 5B, 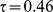), but only a weak and statistically insignificant correlation to phylogenetic profile similarity (Fig. 5D, 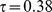 and 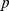-value = 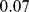). Several interesting observations can be made: First, gold-standard dataset CCSB-binaryGS [1] is consistently in the top 3 networks having higher relative assortativity ratios (Fig. 5A, B, C, and D). Second, Tarassov's dataset has the highest co-localization assortativity ratio – which is consistent with the fact that the PCA method is unique in that it detects *in-vivo* protein interactions within a 8 nanometer distance [12]. AP/MS screens are also *in-vivo*, but there is a key difference: In TAP the interactions are initiated *in-vivo* but detected *in-vitro*, whereas in PCA everything happens *in-vivo* – the proteins never leave the cell even during detection. Third, Ito full is the worst network for relative compressibility as well as for network assortativity while Ito core has consistently higher assortativity and compressibility. Fourth, the WI-PHI network – a network enriched for direct physical interactions – has consistently both higher assortativity and higher relative compressibility than most datasets from which it is derived. It has been argued that interacting proteins need not to be co-expressed, co-localized, or functionally similar [1]. However, it should be noted that even if not all interactions occur between co-expressed, co-localized or functionally similar proteins, a signal must exist when comparing these true interactions with totally random false positives.

To summarize, the above four validation points substantiate our claim that higher network compressibility is a good proxy for overall network quality. Next, we will discuss in detail how the different experimental methods influence the relative compressibility of available large-scale interactomes.

### Relative compression rates of all large-scale interactomes

**Figure 6:**
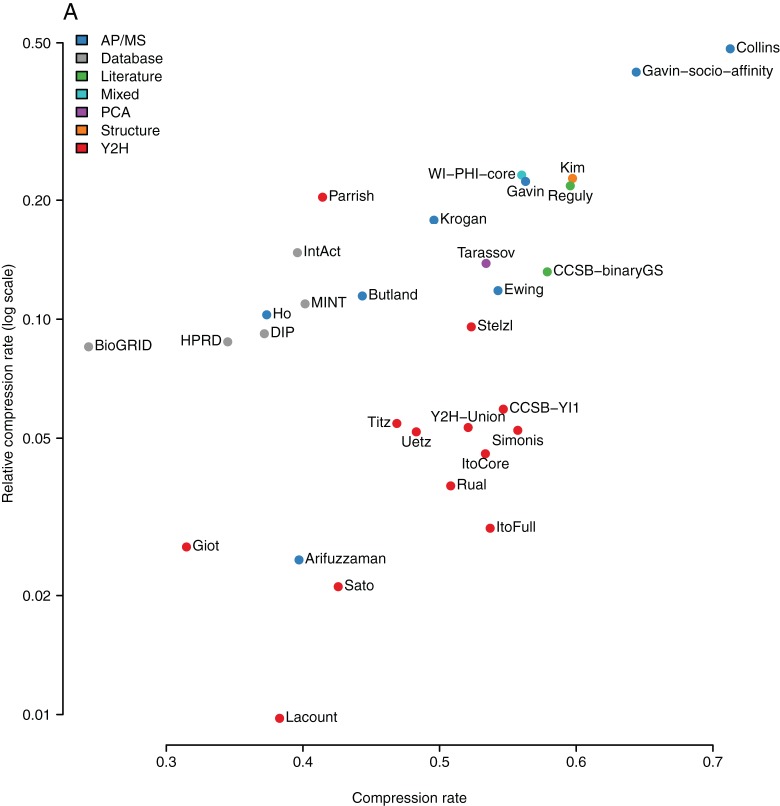
Compression rates and relative compression rates. Relative compression rates plotted against compression rates for several types of large-scale networks: Y2H, AP/MS, PCA, and other derived networks. More details are given in Table 1. *Important note*: by default all AP/MS datasets – except Collins – are interpreted using the *spoke* model. For the Gavin dataset we also add the network derived from socio-affinity scoring.

**Figure 7:**
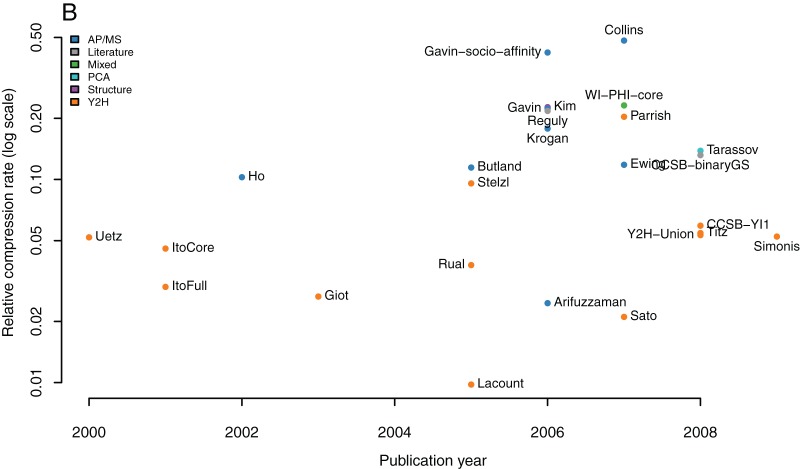
Relative compression rates along time. Progress has been made with higher relative compression rates achieved in recent years.

Table 1 lists the relative compression rates for *all* 22 large-scale interactomes (13 Y2H, 8 AP/MS, 1 PCA), 5 entire databases (BioGRID, IntAct, DIP, MINT, and HPRD), 2 literature curated networks, 1 structural interactome, and 1 mixed dataset (WI-PHI). AP/MS datasets are interpreted using the *spoke* model thus preventing clustering effects – except for the Collins and Gavin datasets that are interpreted using socio-affinity scoring. To prevent a bias in the selection of datasets we defined a strict criteria for what constitutes a *large-scale, unbiased, and symmetric screen* (see Methods for details). Fig. 6 shows a plot of relative compression rates versus absolute compression rates for these networks. Absolute compression rates range from 30% to 70% and relative compression rates from 1% to 48%. Fig. 7 shows that the maximal achieved relative compression rate has been roughly increasing with time, suggesting that progress in the methodologies is leading to networks with increasing richness in patterns and structure.

**Table 1:** Detailed information for figure 6.

author	species	system	year	compression rate	relative compression rate	number of proteins	number of interactions	average degree	PubMed Id
Collins et al.	Yeast	AP/MS	2007	0.71	0.48	1622	9070	11.18	17200106
Gavin et al. (socio-affinity)	Yeast	AP/MS	2006	0.64	0.42	1462	6942	9.50	16429126
Gavin et al. (*spoke*-model)	Yeast	AP/MS	2006	0.56	0.22	1386	3244	4.68	16429126
Krogan et al.	Yeast	AP/MS	2006	0.50	0.18	2708	7123	5.26	16554755
Ewing et al.	Human	AP/MS	2007	0.54	0.12	2294	6449	5.62	17353931
Butland et al.	E. coli	AP/MS	2005	0.44	0.11	1277	5324	8.34	15690043
Ho et al.	Yeast	AP/MS	2002	0.37	0.10	1693	8038	9.50	11805837
Arifuzzaman et al.	E. coli	AP/MS	2006	0.40	0.02	2457	8663	7.05	16606699
Tarassov et al.	Yeast	PCA	2008	0.53	0.14	1507	3030	4.02	18467557
Parrish et al.	C. jejuni	Y2H	2007	0.41	0.20	1326	11659	17.59	17615063
Stelzl et al.	Human	Y2H	2005	0.52	0.10	1664	3083	3.71	16169070
Yu et al. (CCSB-YI1)	Yeast	Y2H	2008	0.55	0.06	1278	1641	2.57	18719252
Titz et al.	T. pallidum	Y2H	2008	0.47	0.05	724	3627	10.02	18509523
Yu et al. (Y2H-Union)	Yeast	Y2H	2008	0.52	0.05	2018	2705	2.68	18719252
Simonis et al.	C. elegans	Y2H	2009	0.56	0.05	1515	1748	2.31	19123269
Uetz et al.	Yeast	Y2H	2000	0.48	0.05	806	644	1.60	10688190
Ito et al. (core)	Yeast	Y2H	2001	0.53	0.05	813	761	1.87	11283351
Rual et al.	Human	Y2H	2005	0.51	0.04	1527	2529	3.31	16189514
Ito et al. (full)	Yeast	Y2H	2001	0.54	0.03	3243	4367	2.69	11283351
Giot et al.	D. melanogaster	Y2H	2003	0.31	0.03	6988	20240	5.79	14605208
Sato et al.	Synechocystis	Y2H	2007	0.43	0.02	1915	3100	3.24	18000013
LaCount et al.	P. falciparum	Y2H	2005	0.38	0.01	1272	2643	4.16	16267556
Aranda et al. (IntAct)	292 species	Database	2010	0.40	0.15	46011	162082	7.05	4,247 publ.
Ceol et al. (MINT)	332 species	Database	2010	0.40	0.11	29407	77954	5.30	2,942 publ.
Prasad et al. (HPRD)	Human	Database	2010	0.34	0.09	9463	35021	7.40	453,521 publ.
Breitkreutz et al. (BioGRID)	15 species	Database	2010	0.24	0.09	29499	229471	15.56	22,645 publ.
Salwinski et al. (DIP)	230 species	Database	2010	0.37	0.09	20685	58596	5.67	3,609 publ.
Reguly et al.	Yeast	Literature	2006	0.60	0.22	1536	2844	3.70	16762047
Yu et al. (CCSB-binaryGS)	Yeast	Literature	2008	0.58	0.13	1090	1263	2.32	18719252
Kiemer et al. (WI-PHI-core)	Yeast	Mixed	2007	0.56	0.23	2443	5244	4.29	17285561
Kim et al. (SIN)	Yeast	Structure	2006	0.68	0.22	1178	2195	3.72	17185604

The complete list of protein interaction networks analyzed is given together with the species, system, publication year, compression rate, relative compression rate, number of nodes and edges, average number of interaction partners (avg. num. of int. partners), and PubMed identifier of publication for referencing. Networks by [89], [90], and [91] are excluded because they are not comparable to the other networks – they are highly asymmetric (see methods section for detailed information on how the networks were compiled). Important note: by default all AP/MS datasets are interpreted using the *spoke* model, but for the Gavin dataset we also add the network derived from socio-affinity scoring.

#### Average signal

To investigate the “average signal” of all available interactome data we computed the relative compression rate of all protein interaction data available in the multi-species databases: IntAct, MINT, BioGRID, and DIP. These database averages cluster around a relative compressibility of 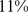. The DIP database covers 8 large-scale datasets whereas MINT covers 11, IntAct covers 20, and BioGRID covers 18. DIP covers interactions from 3,609 publications, BioGRID from 22,645 publications, IntAct from 4,247 publications, MINT from 2,942 publications. We note that the relative compressibility of IntAct is greater than that of MINT, which is greater than that of DIP, HPRD, and BioGRID, this shows that the IntAct database is slightly richer in clique and biclique patterns.

#### Y2H with two-phase pooling has best compression

First introduced by [68], the Yeast two-hybrid system (Y2H) is a widely used technique for protein interaction testing. Applying Y2H for large-scale interactome mapping raises scalability challenges which have been addressed with three approaches: library screens, matrix screens, and the recent smart-pooling screens such as two-phase pooling [69].

Table 2 shows that the two most compressible networks – Stelzl and Parrish – were derived using two-phase pooling Y2H screens, the first having a lower screening completeness than the second. Parrish's network was derived from Campylobacter jejuni, a species with a small genome (1643 coding sequences), and 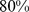 of all proteins were present as baits and preys. A screening completeness of 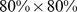 was achieved – 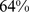 of all protein pairs where screened for interaction. In contrast, Stelzl et al. searched a sizeable but smaller fraction (9%) of the 300 times larger Human interactome search space [70]. This observation suggests that already sensitive screens can deliver interactomes richer in patterns and motifs, if the quadratic size of proteomes can be overcome.

**Table 2:** Strategies for Y2H screening.

datatset	species	strategy	num. of prot. coding genes	screening completeness	avg. num. of int. partners	rel. comp. rate
Stelzl et al.	Human	two-phase pooling (8)	22,286	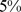	3.7	10%
Parrish et al.	C. jejuni	two-phase pooling (96)	1,685	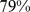	17.5	20%
Titz et al.	T. pallidum	matrix	1,028	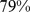	10.0	5%
Rual et al.	Human	library	22,286	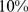	3.3	4%
Simonis et al.	C. elegans	library	20,185	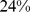	2.3	5%
Giot et al. [110]	D. melanogaster	library	14,144	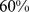	5.7	3%
Yu et al. (CCSB-YI1)	Yeast	library	5,797	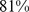	2.5	6%
Ito et al. (core)	Yeast	library	5,797	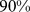	1.8	5%
Uetz et al.	Yeast	library	5,797	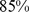	1.6	5%
LaCount et al.	P. falciparum	library	5,268	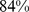	4.1	1%
Sato et al. [109]	Synechocystis	library	3,569	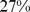	3.2	2%

There are three main strategies for large-scale Y2H screens, briefly: i) matrix – all bait-prey pairs are tested, ii) library – preys are pooled and growing colonies are picked and then sequenced, and iii) two-phase pooling – preys are pooled in a first phase and in a second phase baits that reported interactions are pooled and screened against individual preys (see [69], [87], [88] for reviews). In the Parrish screen pools group 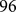 preys compared to 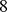 for Stelzl. The *screening completeness* is the proportion of the whole interactome search space that was accessible to the screen: 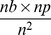 where 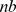 is the number of ORFs cloned for baits, 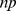 is the number of ORFs cloned for preys and 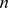 is the estimated number of protein coding genes. In practice, the assay and sampling sensitivity of Y2H screens greatly diminish the effective completeness [73]. For that same reason, screening completeness should not be misconstrued with assay sensitivity – for which the average number of interaction partners is a better indicator.

#### Lower sensitivity of library-based Y2H screens

The lower sensitivity of library based Y2H screens is apparent if one examines the average number of interaction partners. Depending on the database – IntAct, BioGRID, Mint, HPRD, or DIP – the average number of interaction partners per protein can be roughly estimated to be between 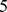 and 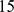. Published estimates similarly range around 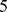 and 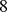 [71]. Interestingly, most library-based Y2H screens exhibit lower values than other strategies. For example, the Titz dataset was derived using the matrix approach for Y2H screening – all bait and prey pairs are tested individually – a potentially more sensitive strategy than library screens [69], [72]. Similarly, two-phase pooling also seems to favor more interaction partners per proteins and thus can be deemed more sensitive.

Overall, Table 2 suggests that differences in relative compressibility between Y2H networks can be partly explained by the different screening strategies and their sensitivities. In contrast, screening completeness has a weaker influence on the relative compression rate than the overall effective sensitivity after taking assay and sampling sensitivity into account [73].

#### AP/MS with knock-in and TAP-tagging has best compression

As Table 3 shows, one AP/MS network – Arifuzzaman et al. – has a low relative compression rate of 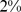 which is below the average for both Y2H and AP/MS datasets [74]. It is also the only screen that uses both cDNA over-expression and the His-tag system instead of maintaining the physiological expression by knock-in tagging [10], and achieving high purity by tandem affinity purification (TAP) [6]. We also observe the higher relative compression rate of Krogan or Gavin (knock-in) versus Ho (cDNA over-expression) in Yeast; and the higher relative compression rate of Butland (knock-in) versus Arifuzzaman (cDNA over-expression) in E. coli [75], [76]. More generally, the two expression modes can be distinguished by the relative compression rate of the corresponding networks (Wilcoxon-Mann-Whitney test with 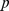-value below 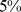).

**Table 3:** Expression modes and tagging systems for AP/MS screening.

datatset	species	expression modes	purification method	num. of prot. coding genes	completeness	rel. comp. rate
Collins et al.	Yeast	physiological expression (knock-in)	TAP	5,797	80%	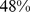
Gavin et al. (socio-affinity)	Yeast	physiological expression (knock-in)	TAP	5,797	78%	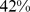
Gavin et al.	Yeast	physiological expression (knock-in)	TAP	5,797	78%	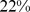
Krogan et al.	Yeast	physiological expression (knock-in)	TAP	5,797	76%	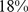
Butland et al.	E. coli	physiological expression (knock-in)	TAP/SPA	4,263	23%	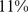
Ewing et al.	Human	over-expression (cDNA)	FLAG-tag	22,286	1%	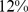
Ho et al.	Yeast	over-expression (cDNA)	FLAG-tag	5,797	10%	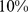
Arifuzzaman et al.	E. coli	over-expression (cDNA)	His-tag	4,263	61%	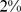

The Arifuzzaman dataset is an outlier when compared with other AP/MS datasets. A possible explanation is that it is the only screen that combined both non-physiological protein expression and His-tagging instead of the superior tandem purification procedure. Note: by default AP/MS datasets are interpreted using the *spoke* model. In addition we list the Gavin network derived by socio-affinity scoring (scores above 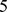). The Collins dataset relies on the same experimental data as the Krogan and Gavin datasets and is derived by a method similar to socio-affinity [7].

#### What is the compressibility of a negative interactome?

What about the compressibility of a network of non-interacting proteins – a negatome? Take a perfectly accurate interactome network and consider its complement or negatome. This network is not random since it contains the same information as the original network: it states exactly which proteins pairs are not interacting. From this the original network can be recovered. Our approach detects this non-randomness in the same way that it detects it for positive interactions. Noise – in the form of both false positives and false negatives – affect both a network and its complement in similar ways by destroying patterns. Our approach does not *know* the difference between positive or negative datasets, but just detects patterns and measures how unlikely they are to appear randomly. We can test this hypothesis by showing that i) relative compressibility of networks and their negation are correlated ii) that a high quality Negatome has high relative compressibility.

As shown in Fig. S8 the relative compressibility of positive and negative networks are correlated (Kendall 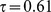, 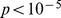). We find a relative compressibility of 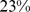 for the Negatome database by [77] in which negative interactions are mined from both literature and protein complex structures. This negative dataset acheives the same level (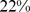) as the positive dataset SIN [78] also derived from structural data. This shows the non-randomness of the Negatome network and in fact hints at its quality.

#### Structure derived interactomes

Since the individual interactions between proteins can directly and unambiguously be extracted from 3D structures, why is the SIN network which is derived from protein complexes' structures ranked below AP/MS networks? This is an artifact of the structural network (SIN) which is derived from structural templates. While the reliability of each interaction is arguably high, the coverage is very sparse and biased for protein and complexes found in solved structures. 3D structures coverage is still order of magnitude lower than coverage achieved by state of the art protein tagging, purification and identification in AP/MS screens which are genome-wide. As shown in validation 1, under-sampling by proteins or interactions leads to decreasing relative compression rates. Therefore if coverage in SIN were to be unbiased and genome-wide it would probably have a higher relative compression rate. Together with PCA, and two-phase Y2H, AP/MS screens produce the networks with the best balance between coverage and accuracy revealing more non-random structures and patterns than other experimental or compilation approaches.

#### How compressible are complete and accurate complex networks?

**Figure 8:**
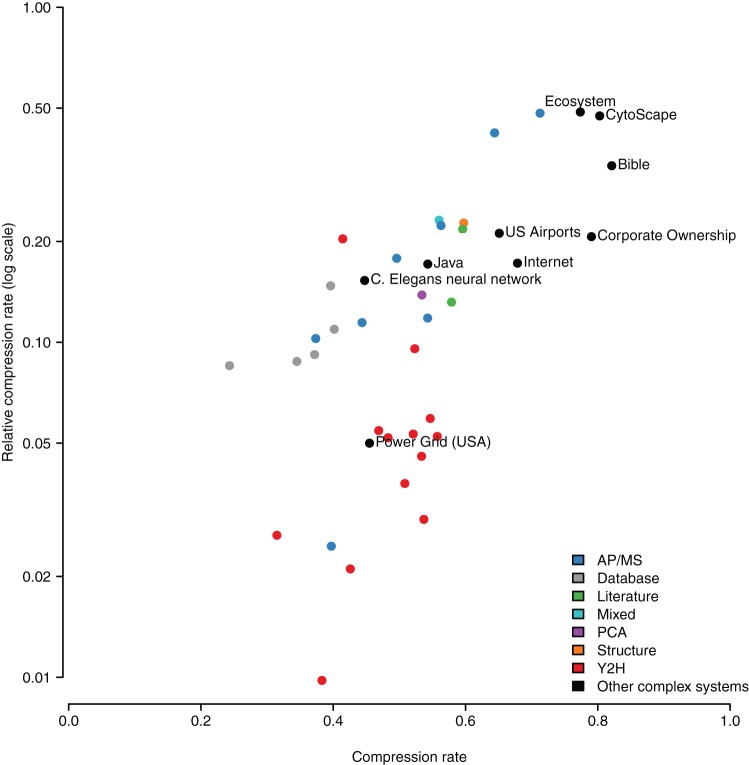
Comparing protein interaction networks with the accurate and complete networks of other complex systems. In order to estimate the relative compression rate of true and complete interactome maps we computed the relative compression rates of a wide range of networks derived from complex systems from ecology, neuroanatomy, software engineering, and the Internet.

In the absence of at least one complete and accurate interactome map it is difficult to estimate the range of true relative compression rates. In particular, an important question is whether some of the high relative compression rates – above 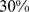 – are a sign of an excess of repetitive patterns and motifs due to systematic errors in the data. To address this point, we compare the relative compressibility of current interactomes with that of accurate and complete networks derived from complex systems of interacting entities. Fig. 8 shows the same plot as Fig. 6A but overlaid with networks such as the C. elegans neural network, Internet, network of North American airports, software module dependency in Java and CytoScape, and others (see methods for complete list and details). In the case of the neural network of C. elegans it should be noted that since the pioneering work of White et al. [79] intensive work has been done to obtain a high confidence neural network. While far from perfect, the level of detail and reproducibility of the observations of synaptic contacts is orders of magnitude more accurate and complete than for any protein interaction network currently available. We observe that all complex systems' networks have a relative compression rate of at least 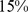 and on average 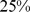. There is one exception: the north American power grid has a relative compression rate of just 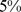. From manual inspection of the different networks, we reached the conclusion that a possible explanation is the network's planarity: it is the only one in which the entities and their interactions are strongly constrained in two dimensions. In the other networks the interacting entities are embedded in higher dimensional spaces and have more freedom to interact – a characteristic shared with protein interaction networks. Fig. 8 suggests that a relative compressibility between 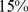 and 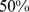 is a signature of networks derived from complex systems whose structure is completely and accurately known. Similar levels of relative compressibility are expected for complete and accurate protein interaction data.

## Discussion

Our results show that experimental methods (AP/MS versus Y2H, pooling strategy, expression level, tagging) strongly influence relative compressibility. Together with the validation steps, this suggests that relative network compressibility is a suitable quality measure for interactomes. However, before drawing this conclusion, there are some more points to consider:

### Organism complexity and relative compression rate

As argued in the introduction, complex and random networks have different topologies. Relative network compressibility can quantify this difference. Can it go further and quantify the *degree* of complexity? If this were the case, then relative network compressibility would be suitable to answer the question whether the difference of complexity of whole organisms is reflected by the difference of the organisms' interactomes network compressibility. More specifically, one would expect a human network more compressible than a mouse or C. elegans network. However, the currently available network data cannot settle this question: Table 2 and 3 show that differences between methods (two-phase pooling versus library, and physiological versus over-expression) have a stronger influence on the relative compression rate than differences in organism complexity as estimated by the ORFeome size. For example, for Y2H networks (Table 2), library screens have relative compression rates around 3% and differ in average by 2 percentage points from each other – independently of the species. In contrast, two-phase pooling screens have higher relative compression rates – above 10%. This shows that any species specific signal is probably hidden by a much stronger method specific signal. Hence, with the current data, network compressibility cannot shed light on the question whether more complex organisms have more complex networks.

### Influence of the network topology on relative compressibility

A reason why network compressibility may not be indicative solely of quality is that it might also reflect changes in other network measures such as network size, degree distribution, or clustering coefficient – which could differ between experimental methods. First we show in Fig. S4 that the relative compressibility is largely independent of the number of proteins or number of interactions. Second, we plot the relative compression rate against average degree and clustering coefficient and consider the relationship for the different types of networks (Y2H, AP/MS, SIN, literature). Some networks (Y2H, literature derived networks) have lower average number of interaction partner per protein than others owing to the experimental method. Can this explain their lower relative compression rates? Fig. S3A shows that the SIN (Kim), PCA (Tarassov), Stelzl, and literature curated networks have similarly low average number of interaction partners and yet have significantly higher relative compression rates. Indeed, we do not observe a significant correlation between average degree and relative compression rate (Kendall correlation 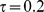 with 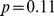). Similarly, networks with low clustering coefficients but high relative compression rates exist (Ho, Ewing [80], Butland, Stelzl). We also observe that the clustering coefficient does not separate Y2H networks from other types of networks as well as does the relative compression rate (Fig. S3B). Indeed, lowly clustered networks can have high relative compression rates because the compression rate captures network motifs based on cliques *and bicliques*. Therefore, bipartite networks that do not contain a single clique – and thus have a clustering coefficient of zero – may still exhibit the whole range of relative compression rates. However, since a part of the compressibility comes from cliques, it is not surprising to observe a significant correlation between clustering coefficient and relative compressibility (Kendall correlation 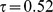 with 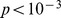). While the average number of interaction partners (average degree) clearly does not explain the whole relative compressibility variability, we further clarify whether there is a causal relationship between relative compressibility and clique content as measured by the clustering coefficient.

### Network de-blurring by clique removal

To better understand the influence of clique content on the relative compressibility we remove from the networks the cliques identified by our algorithm and recompute the relative compression rates and clustering coefficients. Removing the networks' cliques diminishes the proportion of indirect interactions in the network – in a sense it de-blurs the network (see Methods for details). Fig. S5 shows that this more than halves the clustering coefficient of the most clustered networks such as Collins'. However, the relative compression rate remains stable with a median at around 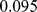 before and after clique removal because i) bicliques play an important role behind compressibility ii) the normalization (see null model in methods section) ensures that the relative compressibility captures non-random occurrences and not just clique and biclique content.

These results confirm that the clustering coefficient is strongly influenced by the amount of cliques while the relative network compressibility is not. As a consequence, network compressibility offers new insights into network topology.

### Differences between Y2H and AP/MS

Overall, we observe that Y2H networks are on average 6 times less compressible than all other networks. AP/MS networks have on average a relative compression rate of 21%, whereas it is 7% for Y2H networks. Wilcoxon-Mann-Whitney tests confirm that the relative compression rate of Y2H is significantly different from PCA, SIN, and literature curated networks (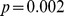) and from AP/MS (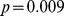). We showed that this difference can be quantified with network compressibility but cannot be captured with classical graph theoretic measures such as average degree or clustering coefficient (Fig. S3). Yu et al. had already noted that Y2H networks had a markedly different topology when compared with AP/MS or literature curated networks [1]. This is in part due to the known lower sensitivity of Y2H screens, which are biased towards binary, transient, and non-cooperative interactions [2], [3]. This particular type of protein interactions do not exhibit as many cliques or bicliques as PCA or AP/MS networks. Possibly, the consistently low average number of interaction partners of Y2H networks indicates that the high selection stringency employed to achieve high specificity leads to sparser networks [2], [3]. Higher relative compressibility levels could be attained with better sensitivity. Our results favor this explanation: recent advances in Y2H screening strategies – in particular two-phase pooling – can bring the relative compressibility of Y2H networks to levels similar to AP/MS and PCA networks (above 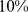 for Stelzl and Parrish datasets). This shows that Y2H networks can be rich in structure and patterns when sensitivity issues are overcome.

While network compression shows some clear differences between types of networks, it should be noted that the method does not rate individual interactions, but it simply measures the structure of the global network. And in this sense the individual interaction of low compression networks are a valuable source of information. Moreover, different experiments (AP/MS, Y2H, PCA) even performed in a perfect world without any noise or artifacts would probably not produce exactly the same networks. Each experiment defining an accessible interactome with different properties (i.e. co-complex versus binary, stable versus transient). Therefore it is conceivable that a perfect and complete Y2H interactome would not be exactly at the same relative compressibility level as a perfect and complete AP/MS or PCA interactome. Comparisons within experimental classes as done in Table 2 and Table 3 are thus easier to interpret than comparisons across experimental classes. In other words, it is better to compare experimental results that measure the same underlying ground truth.

### Example – zooming into chromatin remodeling complexes

As argued by [81], global properties of networks are an average that hides much detail. Therefore, let us consider the patterns underlying compressibility in more detail.

#### Richness in network motifs

**Figure 9:**
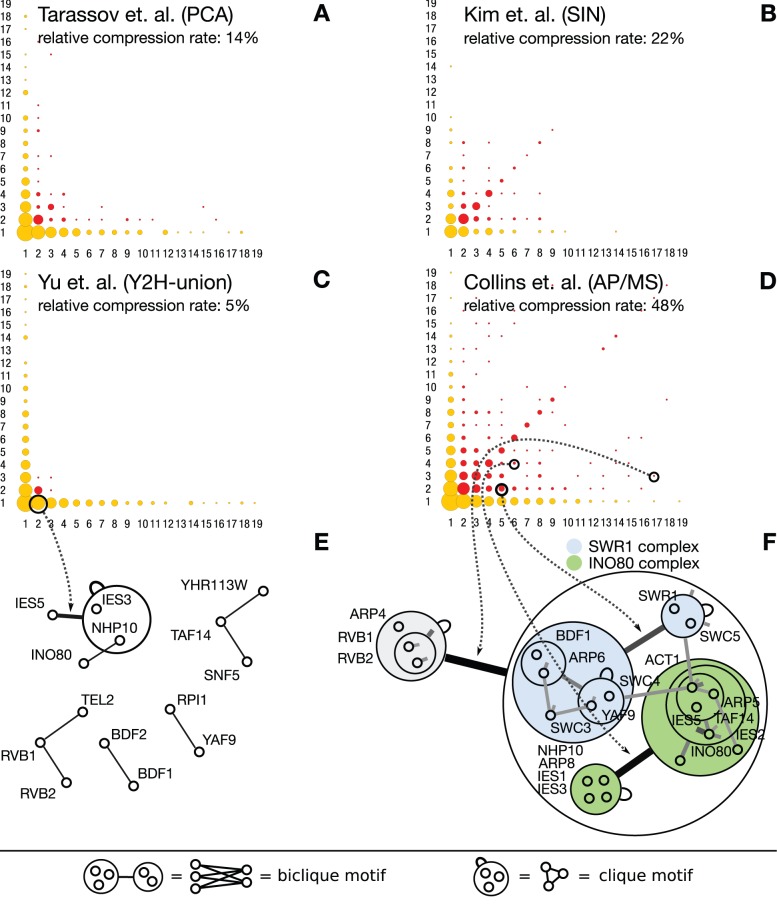
High relative compression rate explained by the richness in network motifs. (**A–F**) The disc charts show the distribution of network motifs – bicliques, cliques and stars – found by power graph analysis. The radius of each disc at a point 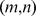 represents – on a log scale – the number of motifs for which 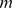 proteins interact with 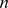 other proteins. Yellow discs correspond to stars, non-diagonal red discs to bicliques, and red discs on the diagonal to cliques or bicliques. High relative compression rate corresponds to denser disc charts and thus to many large cliques and bicliques. (**C**) The Y2H-union network from Yu– which has the highest relative compression rate of all Y2H networks (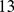 in Table 1) – has a depleted disc chart. (**D**) Collins' AP/MS network has one of the highest relative compression rates and also has one of the densest disc chart. (**E**) The same proteins as in **F** are looked at in the Y2H-union network – only the RVB1/RVB2 sub-complex is visible. (**F**) A modular sub-complex of three essential proteins: RVB1, RVB2, and ARP4 is seen participating in both the INO80 and SWR1 complexes.

Fig. 9A–D show the size and number of motifs obtained from selected networks plotted as disc charts. The number and size of each disc represents the abundance of cliques and bicliques of different sizes. Networks with a high relative compression rate (AP/MS, SIN, PCA; Fig. 9A, B, D) are rich in cliques and bicliques involving many proteins, whereas networks of low relative compression rate (Y2H, Fig. 9C) are depleted.

In particular, panel E and F show a visualization of the low compression Y2H sub-network and the high compression AP/MS sub-network. The panels clearly show that the low compression network has only a few scattered and isolated edges and hence no structure, while the high-compression sub-network comprises non-trivial nested structures.

#### Example – INO80 and SWR1C complexes

Fig. 9F shows an example from the [9] network, which has been confirmed by intense examination in [82]. Here, three proteins – RVB1, RVB2, and ARP4 – interact with 17 other proteins in two chromatin remodeling and DNA repair complexes. RVB1 and RVB2 are the subunits of a hetero-dodecameric DNA helicase [83]. ARP4 is an essential actin-related protein which binds to histone H2A [84]. These three proteins are common subunits in two different complexes: INO80 [85] and SWR1C [86]. While RVB1 and RVB2 constitute an interaction unit as a helicase, they also form a module with ARP4 employed in these two chromatin remodeling complexes. The other 17 components of INO80 and SWR1C are found in the biclique motif. Overall, the modularity of these molecular complexes provides the biological basis for the network's significant compressibility. Some of the interactions between sub-units of the INO80 and SWR1C might be false positives, but these occur between proteins that are in the same complex or that indirectly interact. The effect of these false positives on the compressibility is thus negligible compared to that of true stochastic false positive occurring between otherwise unrelated proteins. In contrast, only the binary interaction between RVB1 and RVB2 is found in the Y2H-union dataset [1].

### Conclusion

Over the past years numerous genome-wide protein interaction datasets have been published. They have been obtained by different experimental methodologies sparking a discussion on data quality and coverage. Since proteomic interactions are inherently co-operative, modular, and redundant, interactomes are expected to be rich in structure and patterns. We propose the relative compression rate as a measure of this richness in patterns and structure and show that it correlates with data quality – understood as encompassing both sensitivity and specificity. We underpin this relationship as follows:

- First, by showing that adding noise (both false positives and false negatives) adversely affects relative compressibility independently of the noise model and type of network.
- Second, gold standard datasets and community-recognized higher quality datasets (low false positive rates) exhibit higher relative compressibility.
- Third, an assessment of confidence thresholds based solely on the relative compressibility agrees with the authors' own benchmarks and analyses aimed at minimizing false positives and false negatives.
- Fourth, we show that relative compressibility correlates with co-expression, co-localization, and shared function.

We also show that well characterized complex systems from other domains also exhibit relative compressibility levels similar to that of many protein interaction networks – thus suggesting that accurate and complete interactomes are also significantly compressible.

We screened all 22 large interactome datasets available, 5 complete interaction databases, as well as four other networks. First, we observe that within an experimental method (Y2H or AP/MS) there is strong effect of the experimental details on the relative compressibility. Networks derived from state-of-the-art purification procedures (Tandem affinity purification, TAP) and detecting interactions of baits expressed at physiological levels (knock-in versus cDNA over-expression) exhibit higher relative compressibility.

Second, we observe that networks derived from Y2H library screens are less compressible than networks derived from two-phase pooling Y2H screens and other experimental methods (AP/MS and PCA). The consistently low average number of interaction partners of networks derived from library Y2H screens suggests that the high selection stringency employed to achieve high specificity leads to too sparse networks [2], [3]. In contrast, recent advances in Y2H screening strategies – in particular two-phase pooling – can bring the relative compressibility of Y2H networks to levels similar to AP/MS and PCA networks (above 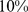 for Stelzl and Parrish datasets). This shows that Y2H networks can be rich in structure and patterns when sensitivity issues are overcome. In fact, more sophisticated “smart pooling” strategies for Y2H screening are being developed and tested such as Shifted Transversal Design and Steiner-triple-system, thus paving the way for higher sensitivity [69], [87], [88].

Based on the results presented in this paper, we make the following recommendations:

- The relative compression rate of new large-scale protein interaction networks can be compared to that of other assays *on the same ground truth* (same species, same interaction space) to estimate overall quality – encompassing both sensitivity and specificity.
- Large-scale interactome screens should employ state-of-the-art methods such as Y2H two-phase pooling and AP/MS with TAP tagging to obtain networks richer in patterns and structure.
- Networks with less than 15% relative compression rate might suffer from poor sensitivity and/or poor specificity.

Overall, relative compressibility is a new measure for comparing networks by their information content defined as the richness in patterns and structure distinguishable from pure noise. This new measure is a good proxy for both sensitivity and specificity and gives complementary information to classic measures such as average degree and clustering coefficient, thus helping to assess the structure of interactomes.

## Materials and Methods

### Network datasets

#### Exhaustive compilation of protein interaction networks

We collected all (21) large-scale protein interaction networks derived from experimental data published between 2000 and 2009. The data files where obtained directly from the supplementary material of the publications. In the cases where the interaction data was not provided in the supplementary material or in the companion website, we obtained the data from one of the interactome databases – Biogrid, Intact, Mint, or DIP. Moreover, we did an automatic scan of these four databases and verified that we had collected all experimental datasets satisfying our strict inclusion criteria: we only consider experimental protein interaction networks that are large-scale and symmetric. We exclude dataset focused on proteins of a specific biological function.

#### Symmetric networks

In symmetric networks the sets of baits and preys are largely overlapping. We exclude highly asymmetric datasets because they are not comparable to symmetric ones. For example, if the number of baits is small in comparison to the number of potential preys. Networks from Formstecher et al. and Rain et al. [89], [90] map interactions around 102 and 261 baits respectively against several thousand preys. Another example is the network by Li et al. [91] which is a highly asymmetric C. elegans protein interactome map between about 2,000 baits and 15,000 preys. This asymmetry introduces a bias in their relative compression rates and makes them incomparable to the other networks (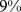 and 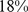 for the Li and Formstecher datasets respectively).

#### Screening completeness

In the case of species with large proteomes such as D. melanogaster, C. elegans, and Human, the screening completeness of individual datasets may be low. However, if the experiment has largely overlapping and symmetric sets of baits and preys – and is unbiased – we included it (for example the Rual [92] and Stelzl [70] datasets).

#### Spoke versus matrix

In the case of AP/MS datasets we interpreted the data using the *spoke* model. For the Gavin dataset we also add the network derived from socio-affinity scoring (binary interactions with a socio-affinity score above 5) for comparison. As explained in [93] *spoke* and *matrix* models are interpretations of protein complex data into binary interactions. In AP/MS screens several preys are identified for a given bait. The *spoke* model assumes that only the preys interact with the bait but not directly with each other – this generally under-estimates the number of interactions as well as ignores any prey-prey interactions. The *matrix* model assumes that any two proteins – preys or baits – interact. Because this model overestimates the number of interactions and introduces many spurious cliques we use the *spoke* model instead.

#### Reference networks

In addition to these experimental networks we added two literature curated datasets [1], [14], and a network derived from protein structures [78]. To estimate the “average” signal of all the interactome data available we also considered the networks derived from the whole protein interaction data compiled in the BioGRID, Intact, Mint, DIP, and HPRD databases. The different species forming distinct and independent connected components of the network – hence giving a species-averaged signal. Finally, we also added the integrated yeast network WI-PHI [61] enriched for direct physical interactions compiled from several datasets: Gavin, Krogan, Ito, Uetz, and BioGRID, BIND, IntAct, and Mint [2], [7], [8], [59], [62]–[65].

#### Overlap between datasets

Some of the datasets overlap: the Ito full dataset contains the same interactions as the Ito core dataset with the addition of lower confidence interactions. The network by Collins et al. [9] is a computational reanalysis of the experimental data by Gavin et al. and Krogan et al. [7], [8] with a similar method to Gavin's socio-affinity. The Y2H-Union dataset [1] is a merge of three high quality Y2H datasets: Ito-core, Uetz and the recent CCSB-YI1 [1], [2], [59]. As mentioned above, the WI-PHI network contains data from most other yeast datasets.

Graphs, power graphs, and compressibility.

#### Graphs

A graph 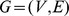 is a set of nodes 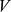 and a set of edges 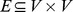 [94], [95]. We consider undirected graphs: 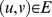 implies 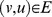. The degree of a node in the network is the number of edges to which it is adjacent.

#### Clustering in protein interaction networks

The notion of clustering or edge-transitivity in networks was first introduced by [96]. Watts et al. defined the network's clustering coefficient as the average local clustering coefficient defined for each node [97]. The clustering coefficient 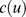 of a node 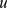 is the proportion of interactions between the neighbors of 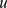 relative to the maximal number of potential interactions:
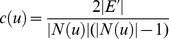



Where 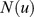 is the neighborhood set of 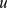 in 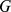, 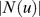 the cardinality of this set, and 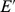 the set of edges in the neighborhood subgraph 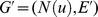. Hence, 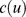 measures how connected is the neighborhood of 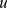 is. If all the neighbors of 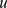 are adjacent then 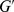 is a clique and 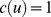, but if none of the neighbors are connected then 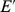 is empty and 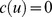.

#### Power graphs

Given a graph 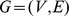 where 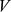 is the set of nodes and 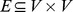 is the set of edges, A *power graph* 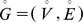 is a set of power nodes 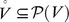 and a set of *power edges* 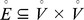. We say that two disjoint (such that 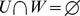) power nodes 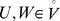 are adjacent if there is a power edge 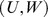 in 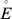. All power nodes in 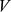 must participate in at least one power edge.

A power graph 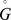 represents graph 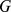 when the following holds: If and only if in 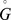 two power nodes 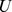 and 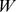 are adjacent, then in 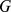 all nodes in 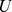 are adjacent to all nodes in 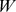:


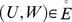
 if and only if 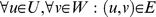


Similarly, if and only if a power node is self-adjacent, then in 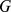 the nodes in 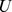 are all adjacent to each other:


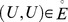
 if and only if 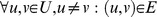


There is one exception, we ignore self-adjacent nodes: 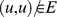. It follows that power edges in 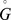 represent bicliques, cliques and stars in 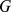. Reciprocally, given a graph 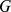, its bicliques, cliques and stars can be represented by power edges in 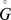. In addition we further constrain the definition of power graphs by requiring the following two conditions:

#### Power node hierarchy condition

Any two power nodes are either disjoint, or one is included in the other. Therefore, power nodes form a hierarchy. This guarantees that the power node hierarchy can be represented in the plane which facilitates visualization.

#### Power edge partition condition

Each edge of the original graph is represented by one and only one power edge. In other terms, the power edges form a partition of the set of edges.

#### Power graph algorithm

The power graph algorithm is described in [56] and an implementation can be found here:

http://www.biotec.tu-dresden.de/schroeder/group/powergraphs.

The algorithm proceeds in two phases: The first phase of the algorithm collects candidate power nodes and the second phase uses those for the search for power edges. In the first phase potential power nodes are identified with hierarchical clustering based on neighborhood similarity. A set of nodes is a candidate power node if its nodes have neighbors in common. We use a hierarchical clustering algorithm based on neighborhood similarity to identify such sets. The similarity of two neighborhoods is the Jaccard index of these two sets. It ranges always between 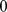 and 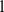: it is 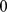 if the sets 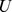 and 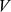 have no common neighbors, and 1 if both have identical neighborhoods. Neighborhood similarity clustering is an intuitive way to identify candidate power nodes. Additional to the hierarchy of sets of nodes achieved with the clustering, to detect stars and other highly asymmetric bicliques in the second phase, we add for each node 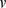 two sets to the candidate power nodes: Its neighborhood set 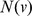 and the set of common neighbors of the nodes in 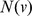, 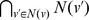, that contains at least 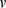.

In the second phase power edges are searched. The minimal power graph problem is to be seen as an optimization problem in which the power graph achieving the highest edge reduction is searched. The greedy power edge search follows the heuristic of making the locally optimum decision at each step with the aim of finding the global optimum. Among the candidate power nodes found in phase one each pair that corresponds to a power edge is a candidate power edge. The candidates abstracting the most edges are added successively to the power graph.

As explained above, power graphs exploit shared neighbors of two proteins as pattern for compression. In principle, any algorithm exploiting shared neighbors should perform similar to power graphs [45]–[52].

#### Clique removal with power graph analysis

For Fig. S5 we have used power graph analysis to filter out cliques from the networks. For each network we compute its corresponding power graph and identify the reflexive power edges that represent cliques in the original network. We then remove from the original network all the interactions corresponding to these cliques. For example, in Fig. 9F the two cliques: ARP4/RVB1/RVB2 and NHP10/ARP8/IES1/IES3 are identified and all 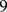 corresponding interactions are removed from the network.

#### Compression rate

Compression rates for protein interaction networks and rewired networks were calculated with the power graph algorithm. The compression rate of a network is calculated from a power graph by computing the edge reduction. If the original network has 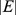 edge and the power graph 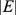 edges, then the compression rate is:
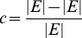



The compression rate is between 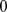 and 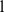. If the power graph has the same number of edges as the original network, then the compression rate is 0. The maximal compression rate is achieved for a completely connected network, which reduces to one power edge.

Clique or biclique membership is not covered in the measure of compression rate because it only assesses the number of edges before and after compression. There are two reasons for our choice: First, simplicity – our goal is to keep the measure as simple as possible. Combining reduction of nodes and edges into one measure leads directly to a number of subsequent questions: Are they of equal importance? Should they be weighted? How should they be combined?

Second, compression with and without nodes strongly correlate. Fig. S6 plots compression rate defined solely on edges *versus* compression rate defined on edges and nodes. The high correlation coefficient of 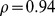 shows that the dominating factor in the compressibility of interactomes are edges and thus nodes can be ignored.

#### Measuring both clique and biclique content

An important point is that compressibility as measured by power graphs can capture network motifs based on cliques but also based on bicliques. Therefore, a bipartite network that does not contain a single clique can still exhibit the whole range of compression rates. Therefore networks with a clustering coefficient of zero may still have high compression rates – see *South Florida ecosystem* network in Table 4.

**Table 4:** Networks of complex system's are compressible.

network	source	year	number of nodes	number of edges	average degree	clustering coefficient	relative compression rate
South Florida Ecosystem	[111]	2000	381	2,137	11.2	zero	0.48
Cytoscape class dependencies	Cytoscape	2009	615	3,463	11.2	0.26	0.47
Bible co-appearance network	[112]	1993	130	743	11.4	0.77	0.33
US Airports	[113]	2007	500	2,980	11.9	0.61	0.21
Corporate Ownership	[114]	2002	7,253	6,711	1.8	0.01	0.20
Java library class dependencies	Java	2006	1,538	7,817	10.1	0.39	0.17
Internet (autonomous systems)	[115]	2006	22,963	48,436	4.2	0.23	0.17
C. elegans neural network	[79]	1986	297	2,148	14.4	0.29	0.15
Power Grid (USA)	[97]	1998	4,941	6,594	2.6	0.08	0.04

Network relative compressibility in the range 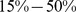 is typical of complete and accurate networks derived from complex systems. Note: the *South Florida Ecosystem* network has a clustering coefficient of zero because it is a *strict* bipartite network – the relative compressibility is not solely measuring clique content and clustering in networks.

#### Relative compression rate

The relative compression rate measures an original network's compression rate in relation to an average random network of same topology. To compute the relative compression rate one generates 1000 random networks following the null model (see below) and computes the average compression rate. The relative compression rate measures by how much the original network's compression rate differs from the average random compression rate:
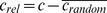



Where 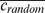 is the mean of the compression rates for the random networks. For example, a relative compression rate of 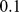 means that the compression rate is 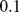 – 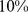 points – higher than the average compression rate of equivalent random networks. The relative compression rate is a more relevant measure than the compression rate because a certain level of compressibility is always expected, even from random networks. Fig. S7 shows the compression rates plotted against the average compression rates of randomly rewired networks having the same number of nodes, edges and same degree distribution.

### Random networks and network noise

#### Network null model – degree preserving random rewiring

Given a protein interaction network, we generate a large (1000) population of randomly rewired networks. These random networks have the same number of nodes and edges, as well as the same number of interaction partners per node and hence the same degree distribution as the original network. These networks are generated by randomly re-wiring the original network [98]. Two randomly chosen interactions A–B and C–D are replaced by two new interactions A–C and B–D. This preserves the number of edges per node. This operation is repeated a number of times which is a multiple of the number of edges in the network – thus ensuring that almost all edges are rewired at least once. Moreover, each random network is generated from a previously rewired network and thus correlation with the original protein interaction network is unlikely.

#### Models for false negatives and false positives

For all noise models we perturb the original networks and recompute their compressibility. For the results in Fig. 2 we used two models for false positive and false negative interactions. The first model – ER for Erdös–Rényi – consists in randomly adding or removing interactions. The interaction partners are drawn from a uniform distribution over all proteins following the exponential model first described by Erdös and Rényi [57]. The second model – BA for Barabási-Albert – consists in randomly removing interactions from poorly connected proteins and randomly adding interactions to highly connected proteins. Interaction-rich proteins get richer and interaction-poor proteins get poorer. The interaction partners are drawn from a distribution in which the probability for each protein is proportional (or inversely proportional) to the number of its interaction partners [58]. For both models we analyzed the influence of false positives (added interactions) and false negatives (removed interactions) separately, thus leading to four different models: ER false negatives, ER false positives, BA false negatives, BA false positives. *Important note:* since we consider symmetric large-scale screens where the set of baits is largely overlapping to the set of nodes, we don't need to consider the bait or prey status of proteins in our noise models. Moreover, it should be noted that due to the incompleteness of current interactome data, removed interactions are sometimes false positives, and added interactions are sometimes false negatives. For the results in Fig. 3A we randomly remove proteins using a uniform distribution on the network's proteins. For Fig. 3 we construct a probability distribution based on the network's degree distribution. The degrees of added proteins are drawn from this distribution thus preserving the topology of the original network. The added proteins are *cloned* from existing proteins in the networks and their connections are randomly rewired while preserving the number of neighbors. This minimally affects the degree distribution and introduces proteins in the network that have completely random interactions.

#### Analysis of false negatives and false positives' influence on the relative compressibility

We generated networks with simulated false positives and false negatives for 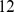 Yeast protein interaction networks. For each of the four models we considered 30 different levels of false positives and negatives from 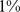 to 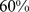 – in total 1,440 networks. For each of these 1,440 networks we generated 1,000 networks having the same number of nodes, edges and same degree distribution. More than 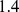 million compression rates were computed requiring 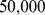 CPU-hours on a 2,500 CPUs supercomputer.

### Correlations

#### Correlating interaction confidence scores with relative compressibility

We obtained the raw interaction confidence scores for the three datasets by Gavin et al., Parrish et al., and Tarassov et al. (provided in the supplementary material of the publications). As illustrated on Fig. 4A, we extracted sub-networks by selecting interactions with confidence scores within a given minimal and maximal value. To each pair 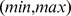 corresponds a sub-network for which we computed the compression rate. The relative compression rate was obtained as the difference between the compression rate of each sub-network and the compression rate of the whole network after randomization (see procedure described previously). In this context, the compressibility is measured relative to the random baseline compressibility of the whole network. This is required because otherwise sub-networks richer than the whole network in motifs and patterns would not be detected. Cells close to the diagonal represent small confidence intervals and thus correspond to small sub-networks. Unfortunately, few publications offer the raw unfiltered interaction data with confidence scores – we agree with Hart et al. that a wider availability of such raw data would greatly benefit new analysis on error rates [99].

#### Correlation of network compressibility with co-expression, co-localization, shared function, and phylogenetic similarity

We correlate interactions with gene co-expression, cellular function, cellular co-localization, and phylogenetic profile similarity for 12 Yeast networks and for all interacting pairs of proteins for which we have complete information. We use the following assortativity ratio:
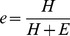



Where 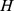 is either the number of homotypic interactions for which the proteins are significantly co-expressed, share a cellular function, are found in at least one common cellular compartment, or have significantly similar phylogenetic profiles. 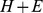 are all the interactions – homotypic and heterotypic – for which we have complete information about *both* interacting proteins. We use data compiled by [100] for defining co-expression and phylogenetic similarity. Gene co-expression data is computed by correlating mRNA gene expression profiles obtained from 497 microarray experiments [100]. Phylogenetic similarity is calculated by correlating phylogenetic profiles which are strings that encode the presence or absence of a protein in every known genome [101]. We consider that two proteins are co-expressed if they have a log-likelihood score above 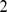, and phylogenetically similar if the log-likelihood score is above 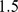. Shared function was measured using the Gene Ontology (GO) molecular function (MF) and biological processes (BP) annotations as provided by the SGD database [102]. For co-localization, we use the genome-wide protein localization data from [103]. Two proteins are co-localized if they share at least one cellular compartment, and two proteins share cellular function if they have at least one common GO term (BP or MF). As for the relative compression rate we normalize these assortativity ratios by subtracting the average proportion found for equivalent randomized networks. We thus compute the *relative* assortativity ratio:
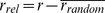



Where 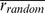 is the mean ratio obtained for randomly rewired networks having the same number of nodes, edges and same degree distribution (see above for network null-model). In Fig. 5 the x-axis is 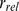 (relative assortativity ratio) and the y-axis is 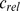 (relative compression rate). Moreover, the reported magnitude and statistical significance of correlations are calculated using Kendall's method (*Kendall* package in R).

### Networks of complex systems

We collected nine networks from the network science literature derived from complex systems of interacting entities (Table 4). These networks were chosen for their accuracy and completeness: the Internet network, software module dependencies in Java and Cytoscape, North American airport network, ownership relationships of American corporations, a food web in South Florida, co-appearance relationships between characters in the Bible, North American power grid network, and the neural network of C. elegans (the latter has been completely and accurately mapped because of its small size).

## Supporting Information

Figure S1
**Slicing the Kiemer et al. network.** We observe that the sub-network with the most interactions and the highest relative compression rate is found for a minimum socio-affinity score between a minimum of 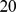 and a maximum of 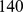. This is in agreement with the authors definition of WI-PHI *core* as having a score above 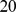 – which corresponds to interactions present in at least 2 datasets as seen in Fig. 4A from [61].(EPS)

Figure S2 **Details for Ito core and full networks.** Data points (dots) are smoothed into curves (line) with the LOWESS (locally weighted scatterplot smoothing) regression method [107]. Removing interactions according to the (**A**) ER noise model and (**B**) BA noise model.
(EPS)

Figure S3
**Low average number of interaction partners is no reason for low relative compression rates.** (**A**) While low relative compression rates imply low average number of interaction partners, low average number of interaction partners does not imply low relative compression rates. Note that the CCSB binary interaction gold standard (CCSB-binaryGS) has a similar average number of interaction partners as most Y2H networks and yet it has a higher relative compression rate. (**B**) **Relative compression rate versus clustering coefficient.** Similarly to the average number of interaction partners, we observe that a low clustering coefficient do not imply a low relative compression significance. For example, the LaCount [108] dataset has a similar clustering coefficient (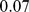) to the Butland dataset (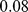), and yet they differ in relative compression rates (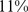 difference). We also observe that the relative compression rate is better than the clustering coefficient at discriminating different screening methodologies.(EPS)

Figure S4 **Relative compression rate versus the number of proteins and interactions in the networks.** The relative compression rate is independent of both the number of nodes and number of edges.
(EPS)

Figure S5
**Clique removal and its effect on the relative compression rate and clustering coefficient.** (**A**) Compression rate versus relative compression rate after clique removal. Removing cliques does not significantly alter the plot from Fig. 6. (**B**) Relative compression rate versus clustering coefficient after clique removal. The same scaling is used for easy comparison to Fig. 13B. The clustering coefficient is drastically reduced for highly clustered networks such as Collins, Gavin socio-affinity (but not Gavin interpreted with *spoke* model), WI-PHI, CCSB binaryGS, and Reguly. For example, Collins' clustering coefficient is more than halved from 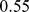 to 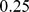. Other networks experience a lesser decrease in clustering coefficient. In contrast, the relative compression rate remains stable in comparison – for example Reguly's stays at 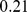, and Titz at 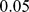.(EPS)

Figure S6 **Edge reduction based on power edges compared to edge reduction based on power nodes and power edges.** We chose the simplest definition of compression rate: we compare the number of edges after and before compression. Counting power edges (after compression) is sufficient because power edges include the information about the two sets that are connected. As shown above, considering power nodes in addition to power edges does not significantly change the compression rate.
(EPS)

Figure S7 **Compression rate versus the average compression rate of randomly rewired networks of same topology.** The relative compression rate is computed by taking the difference between the absolute compression rate and the average compression rate of randomly rewired networks with the same topology.
(EPS)

Figure S8
**Relative compressibility correlation between positive and negative networks.** Inverted networks are listed together with the number of nodes, number of edges and average degree, corresponding relative compressibility, and relative compressibility for original positive network. The relative compressibility of positive and negative networks are correlated (Kendall 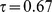, 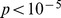). We find a relative compressibility of 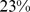 for the Negatome database by [77]. Important note: the negative Negatome is a network of positive interactions.(EPS)

Figure S9
**Overlap between the subsets of Yeast proteins screened by Y2H, AP/MS, and PCA.** In total, 3507 Yeast proteins were found to interact at least once by any of the three methods. Only 287 proteins were found by all methods to be part of an interaction. (**B**) Common protein interaction space. Between the 287 proteins explored by all methods, 500 interactions were reported in at least one experiment. Only 42 interactions were confirmed by all three methods. (**C**) Enrichment analysis of the common protein interactions space. Following the Venn diagram B, we show enriched MIPS annotations for proteins participating in interactions specific to each method (Y2H, AP/MS, and PCA) and common to all (intersection). The number in each box is the p-value majoring exponent for the enrichment (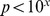).(EPS)
